# Cas9 Mouse Model of Skull Base Meningioma Driven by Combinational Gene Inactivation in Meningeal Cells

**DOI:** 10.1111/cns.70287

**Published:** 2025-02-25

**Authors:** Hailiang Tang, Feng Xu, Dan Sun, Lingyang Hua, Ji Xiong, Ming Xu, Jian Xu, Ping Zhong

**Affiliations:** ^1^ Department of Neurosurgery, Huashan Hospital Fudan University Shanghai China; ^2^ Department of Operation Center, Huashan Hospital Fudan University Shanghai China; ^3^ Department of Pathology, Huashan Hospital Fudan University Shanghai China

**Keywords:** Cas9 mouse model, *Nf2* gene, skull base meningioma

## Abstract

**Introduction:**

Neurofibromatosis type 2 (*Nf2*) gene inactivation is common in sporadic and *Nf2*‐related meningioma. There is currently scant literature describing the development of an intracranial meningioma model in animals. Given the role of *Nf2* and other gene inactivation in meningeal cells, we used Cas9 mice here as the background host to establish a new animal model of skull base meningioma in this study.

**Aims:**

Cas9 transgenic mice were purchased from Jackson Laboratory and raised in our institution. Subsequently, meningeal cells were obtained from the Cas9 transgenic mice, cultured in medium, and passaged in vitro. We then prepared lentivirus vector pLentiCre/gRNA, which could express the elements blocking the function of four genes: *Nf2, P15* ^*Ink4b*^, *P16* ^*Ink4a*^ *,* and *P19* ^*Arf*^. We infected the meningeal cells with the lentivirus vector pLentiCre/gRNA and tested the expression of these four genes in those infected meningeal cells. Next, adeno‐associated virus vector pAAVCre/gRNA was injected in vivo into the skull base meningeal cells of the neonate Cas9 transgenic mice. These mice were observed once a week and killed 10 months later for brain inspection and pathological analysis.

**Results:**

Twenty Cas9 transgenic mice were successfully bred. Five mice were killed so that meningeal cells could be extracted, cultured, and infected with the lentivirus vector pLentiCre/gRNA for 72 h in vitro. The gene function test showed that *Nf2, P15* ^*Ink4b*^, *P16* ^*Ink4a*^ *,* and *P19* ^*Arf*^ were all blocked in the infected meningeal cells, which indicated that the lentivirus vector pLentiCre/gRNA could effectively block the expression of the four genes in targeted cells. Then pAAVCre/gRNA was injected into the skull base meningeal cells of 15 mice in vivo, and nine mice were observed for 10 months so that the intracranial tumor growth could be assessed. Among these nine mice, pathological analysis showed that six mice had benign meningioma subtypes similar to human meningioma, one mouse had atypical meningioma, one mouse had malignant meningioma, and one mouse had sarcoma.

**Conclusions:**

The Cas9 mouse model of skull base meningioma generated with the *Nf2* genetic defect and the combinational loss of *P15* ^*Ink4b*^, *P16* ^*Ink4a*^, and *P19* ^*Arf*^ could provide a new tool for investigating the pathogenesis of meningioma and the development of chemical interventions for this disease.

## Graphical abstract

Sketch of Cas9 transgenic mouse model for skull base meningioma.

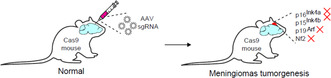

Adeno‐associated virus (AAV) vector pAAVCre/gRNA was introduced into the meningeal cells of the Cas9 mouse skull base. Cas9 functions were activated by Cre in these cells and caused mutations of the four genes (Nf2, P15Ink4b, P16Ink4a, and P19Arf) in meningeal cells, which could lead to tumorigenesis of meningioma in the skull base of Cas9 mice.

## 1 Introduction

Intracranial meningioma, which arises from the arachnoidal cells of the leptomeninges that cover the brain, accounts for approximately 30% of all central nervous system (CNS) tumors [1]. Most meningiomas are slow‐growing brain tumors with benign characteristics, are categorized as grade I by the World Health Organization (WHO) classification, and are associated with a favorable prognosis after surgical resection. However, about 20% of meningiomas are malignant and are categorized as WHO grade II or grade III, indicating biological aggressiveness and a high recurrence rate [2]. Currently, surgical resection and adjuvant radiotherapy are the standard therapies for malignant meningioma, but no effective chemotherapy has been developed. Therefore, it is critical to clarify the mechanisms underlying the development of meningioma and the factors driving its malignant progression or recurrence at the molecular level. This would aid in developing new chemotherapy strategies for treating these refractory meningiomas.

Molecular genetic studies into meningioma have identified Neurofibromatosis type 2 (*Nf2*) as one of the most commonly mutated genes in meningiomas. In addition to *Nf2*, other mutations have also been identified, such as *TRAF7*, *KLF4*, and P16, which are related to the malignant progression of meningiomas [3, 4, 5]. Currently, only a few studies have examined the development of an intracranial meningioma model in animals [6, 7], but such a model would be integral to characterizing the molecular mechanisms of meningioma in vivo.

Most animal models of meningioma described thus far have been mainly based on the implantation of human meningioma cells into immunocompromised mice [8, 9, 10] or on the direct implantation of human meningioma tissue. However, these models cannot mimic the natural development of meningioma, and thus the construction of an in vivo model system in which meningioma arises from normal arachnoidal cells would be highly valuable. In this study, we developed a novel mouse model of skull base meningioma based on *Nf2* gene inactivation. We used Cas9 transgenic mice as the host and then injected adeno‐associated virus (AAV) vector pAAVCre/gRNA, which could block the functions of four genes (*Nf2, P15* ^*Ink4b*^, *P16* ^*Ink4a*^, and *P19* ^*Arf*^), into the meningeal cells of the mouse skull base. After 10 months of observation, the meningioma samples of the mice were examined and sent for pathological analysis.

## 2 Materials and Methods

### 2.1 Preparation of Meningeal Cells From Cas9 Transgenic Mice

Twenty mice of Rosa26‐LSL‐Cas9 knockin were purchased from Jackson Laboratory (Strain #:024857, USA) and successfully bred in our lab. All of the protocols were approved by the Experimental Animals Ethics Committee of Fudan University (2022JS‐318). Five neonatal mice (postnatal day 2) were killed, meningeal tissues were isolated, and single‐cell primary cultures were obtained via mechanical and enzymatic methods. Primary meningeal cells were passaged for four to five generations.

### 2.2 Transfection of Meningeal Cells From Cas9 Transgenic Mice

The lentiviral vector pLentiCre/gRNA was packaged, and the viral expression strategy was as follows: CMV‐NLS‐Cre‐3FLAG‐polyA‐U6‐gRNA1‐U6‐gRNA2‐U6‐gRNA3‐U6‐gRNA4. From this, four gRNAs were screened and separately directed to the *Nf2, P15* ^*Ink4b*^, *P16* ^*Ink4a*^, and *P19* ^*Arf*^ genes.

The titer of the packaged virus was greater than 10^8^/ml. According to a virus: cell ratio of 200:1, the primary meningeal cells were infected, cultured, and passaged. The green fluorescent protein (GDP) signal was observed after 3 days of infection with the pLentiCre/gRNA virus, while no signal was observed in the control group. This indicated that the virus could infect the cells and express Cre, which in turn would activate Cas9 expression. In addition, we found that primary meningeal cells infected with the no‐load virus could no longer be propagated after four passages, while the cells infected with pLentiCre/gRNA exhibited a vigorous growth trend, requiring passaging every 2 days, with no slowing in growth for more than 10 generations.

### 2.3 Target Gene Expression After Viral Infection in Meningeal Cells

After being infected with the pLentiCre/gRNA virus, the meningeal cells were passaged for 10 generations, and some meningeal cells were used for DNA extraction. Polymerase chain reaction (PCR) and sequencing were performed on the site to be edited and revealed that the editing sites set in the meningeal cells for the four target genes (*Nf2, P15* ^*Ink4b*^, *P16* ^*Ink4a*^, and *P19* ^*Arf*^) were all mutated. This indicated that our strategy can indeed simultaneously achieve mutations in the four genes in meningeal cells.

### 2.4 Viral Injection Into the Meningeal Site of Neonatal Mice

Usually, AAV has a high packaging titer and has a long‐lasting effect in vivo [7]. Therefore, according to the pLentiCre/gRNA strategy, we constructed an AAV vector of pAAVCre/gRNA, which could import Cre and the four gRNAs. After the above‐mentioned primary meningeal cells were infected with AAV in vivo, the fluorescence expression was observed as well, indicating that the AAV could activate Cas9 expression as the pLentiCre/gRNA virus. The structure of the pAAVCre/gRNA was as follows: ITR‐U6‐sgRNA(Nf2)‐U6‐sgRNA(p16Ink4a)‐U6‐sgRNA(p15Ink4b)‐U6‐sgRNA(p19Arf)‐pEFS‐Rluc‐2A‐Cre‐shortPA‐ITR.

The prepared pAAVCre/gRNA was injected into the skull base at the subdural position of 2‐day‐old Cas9 transgenic mice, with each mouse being injected with about 10^8^ VG/ml of virus. The mice that survived the injection continued to be observed (one mouse died of viral injection). Two months after injection, five mice were killed, and the expressions of GFP in the injection site at the skull base were observed under microscopy.

### 2.5 Observation of Meningioma Formation by Brain MRI Scan

After 10 months' observation, five mice were sent for a brain magnetic resonance image (MRI) scan in order to detect intracranial tumor growth. This study was conducted on a Bruker 7.0 T scanner (Bruker BioSpin GmbH). The specific method is as follows: Inhale 1%–2% isoflurane (IsoFlo, USA) into mice, and after anesthesia, the mice were placed in a magnetic resonance room for scanning. First, we performed rapid acquisition with relaxation enhancement echo scans in three directions and then performed conventional spin echo sequence, T1‐weighted, and T2‐weighted scans on the entire brain. The scanning parameters are as follows: relaxation time 4000 milliseconds, echo time 15 milliseconds, field of view 25.6 × 25.6 mm, matrix 256 × 256, slice thickness 0.8 mm, scan time 3 min 20 s. After that, Gd‐DTPA (Magnevist, Germany) magnetic resonance enhancer was injected intraperitoneally into mice; the dose was 0.8 mL/kg for enhanced scanning.

### 2.6 Histological and Immunohistochemical Analysis

After 10 months' observation, finally, nine mice tumor specimens were processed for histological examination via standard hematoxylin and eosin (HE) staining and also examined for EMA, vimentin, and Ki‐67 (as detected by MIB‐1 antibody) via immunohistochemical (IHC) staining. The primary antibodies were all obtained from Abcam.

## 3 Results

### 3.1 Transfection of Meningeal Cells From Cas9 Transgenic Mice

The lentiviral vector pLentiCre/gRNA was packaged, and the viral expression strategy was as follows: CMV‐NLS‐Cre‐3FLAG‐polyA‐U6‐gRNA1‐U6‐gRNA2‐U6‐gRNA3‐U6‐gRNA4. From this, four gRNAs were screened and separately directed to the four genes *Nf2, P15* ^*Ink4b*^, *P16* ^*Ink4a*^, and *P19* ^*Arf*^.

**FIGURE 1:**
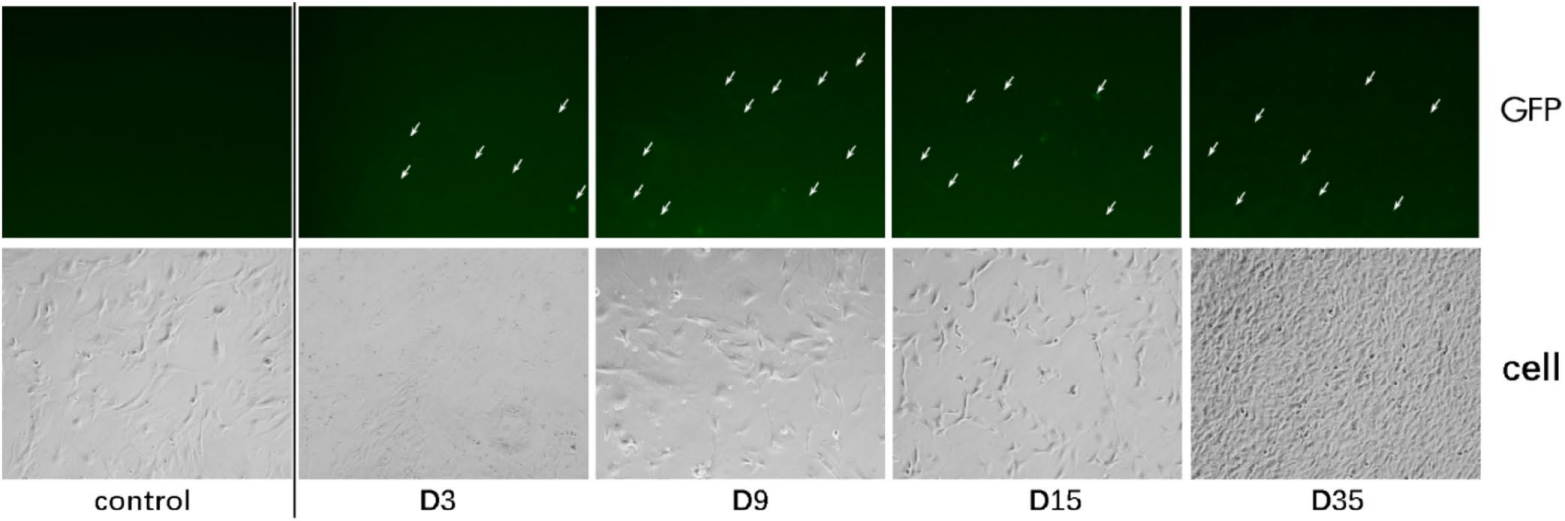
Cas9 was expressed (GFP positive) in infected meningeal cells (D3‐35, 3–35 days after viral transfection), while no expression was observed in the control group.

The titer of the packaged virus was greater than 10^8^/ml. According to a virus: cell ratio of 200:1, the primary meningeal cells were infected, cultured, and passaged. The green fluorescent protein (GDP) signal was observed after 3 days of infection with the pLentiCre/gRNA virus, while no signal was observed in the control group. This indicated that the virus could infect the meningeal cells and express Cre, which in turn would activate Cas9 expression (Figure 1). In addition, we found that primary meningeal cells infected with the no‐virus load could no longer be propagated after four passages, while primary meningeal cells infected with pLentiCre/gRNA exhibited a vigorous growth trend, requiring passaging every 2 days, with no slowing in growth for more than 10 generations.

### 3.2 Targeted Gene Mutation After Viral Infection in Meningeal Cells

**FIGURE 2:**
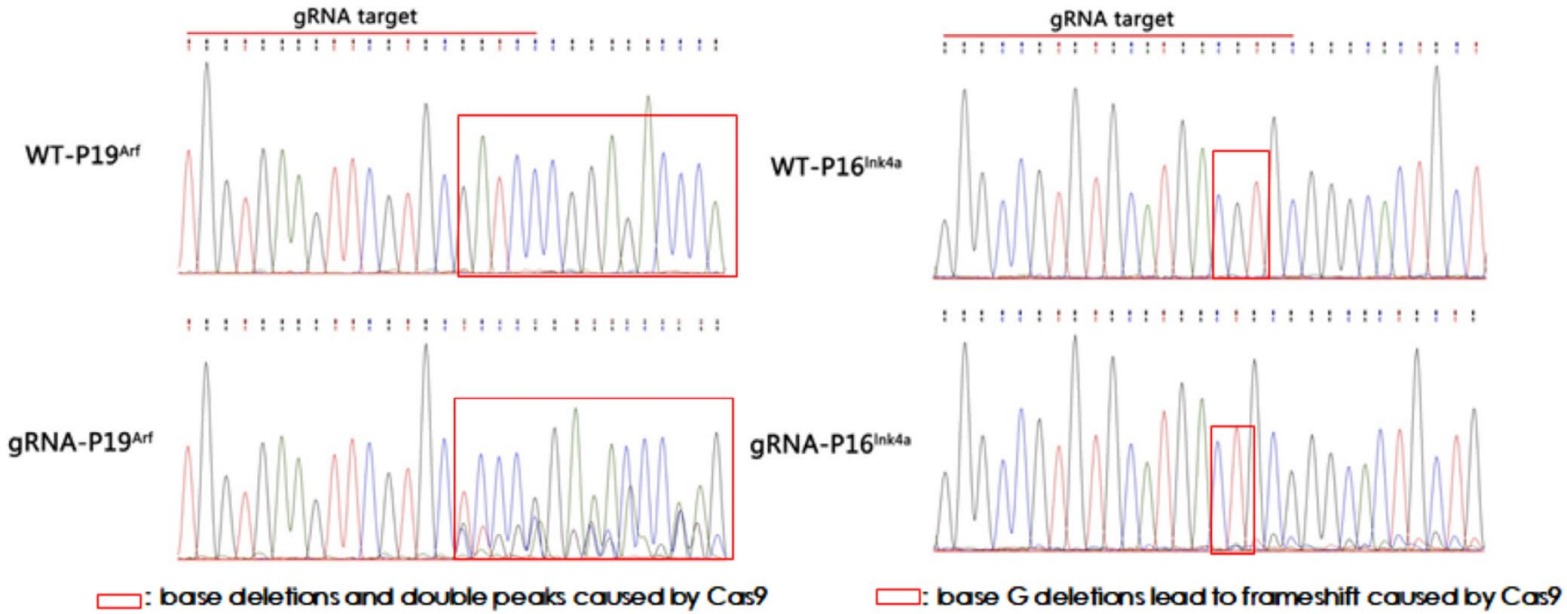
Targeted gene mutations in meningeal cells by Cas9 (p19 and p16).

**FIGURE 3:**
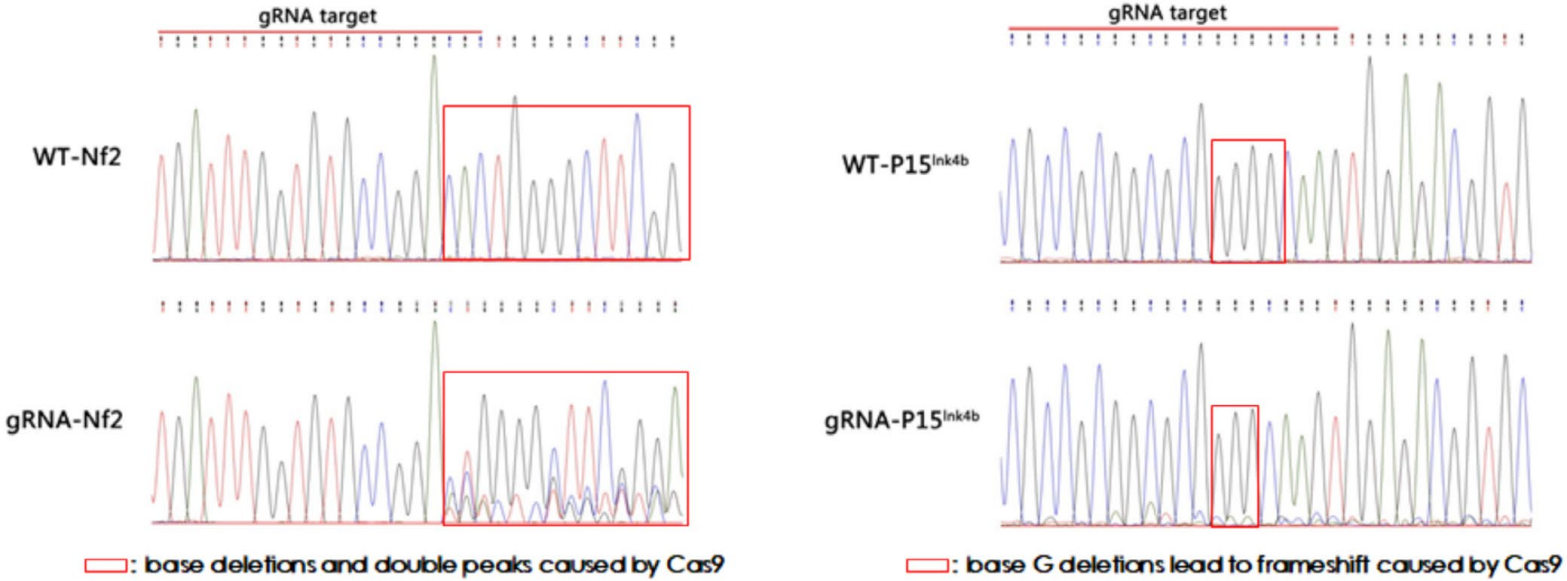
Targeted gene mutations in meningeal cells by Cas9 (Nf2 and p15).

After being infected with the pLentiCre/gRNA virus, the meningeal cells were passaged for 10 generations, and some cells were used for DNA extraction. Polymerase chain reaction (PCR) and sequencing were performed on the site to be edited and revealed that the editing sites set in the cells for the four target genes (*Nf2, P15* ^*Ink4b*^, *P16* ^*Ink4a*^, and *P19* ^*Arf*^) were all mutated (Figures 2 and 3). This indicated that our strategy could indeed achieve mutations in the four genes in meningeal cells simultaneously.

### 3.3 Viral Injection Into the Meningeal Site of Neonatal Mice

**FIGURE 4:**
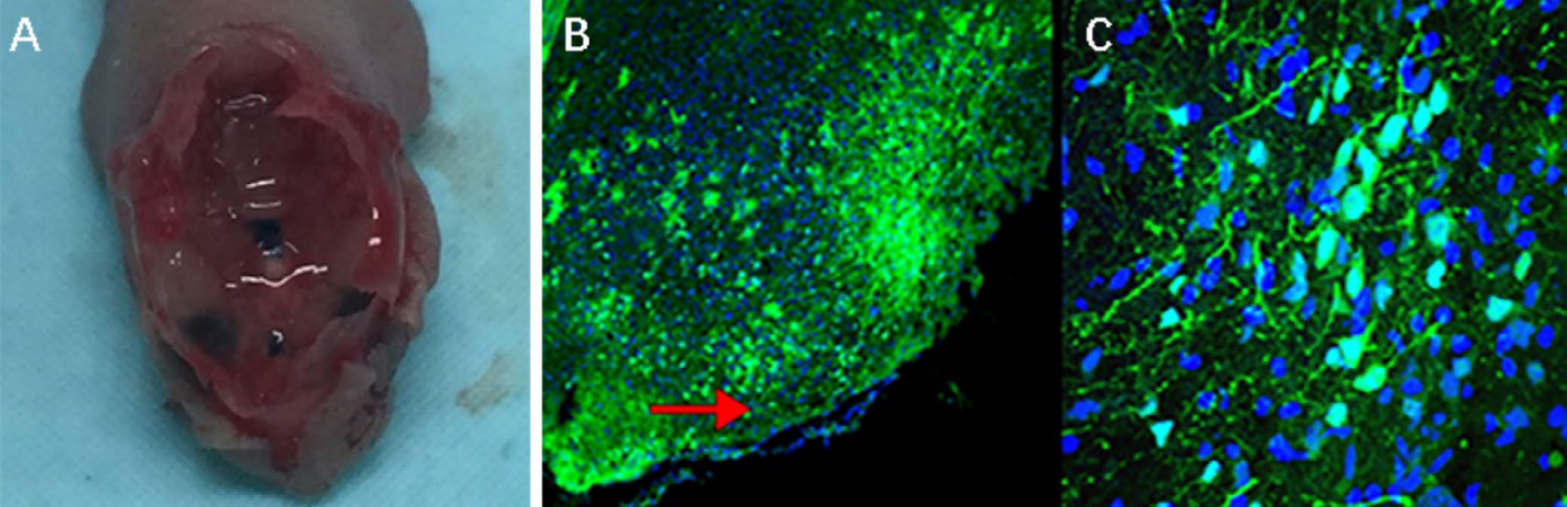
Viral injection into the meningeal site of neonatal mice. (A) Injection of pAAVCre/gRNA into the skull base of Cas9 transgenic mice (P2), and inspection of the injection sites (blue dots) after removing brain tissue. (B‐C) GFP‐positive cells (green) were detected by fluorescence in skull base cells (red mark indicated meningeal areas).

Then we constructed an AAV vector of pAAVCre/gRNA, which could import Cre and the above four gRNAs as well. The prepared pAAVCre/gRNA was injected into the skull base at the subdural position of 2‐day‐old (P2) Cas9 transgenic mice (Figure 4A), with each mouse being injected with about 10^8^ VG/ml of virus. The mice that survived the injection continued to be observed (one mouse died of viral injection). Two months after injection, five mice were killed for inspection, and the expression of GFP fluorescence in the injection site at the skull base was observed (Figure 4B,C).

### 3.4 Detection of Meningioma Formation in Mice by Brain MRI Scan

**FIGURE 5:**
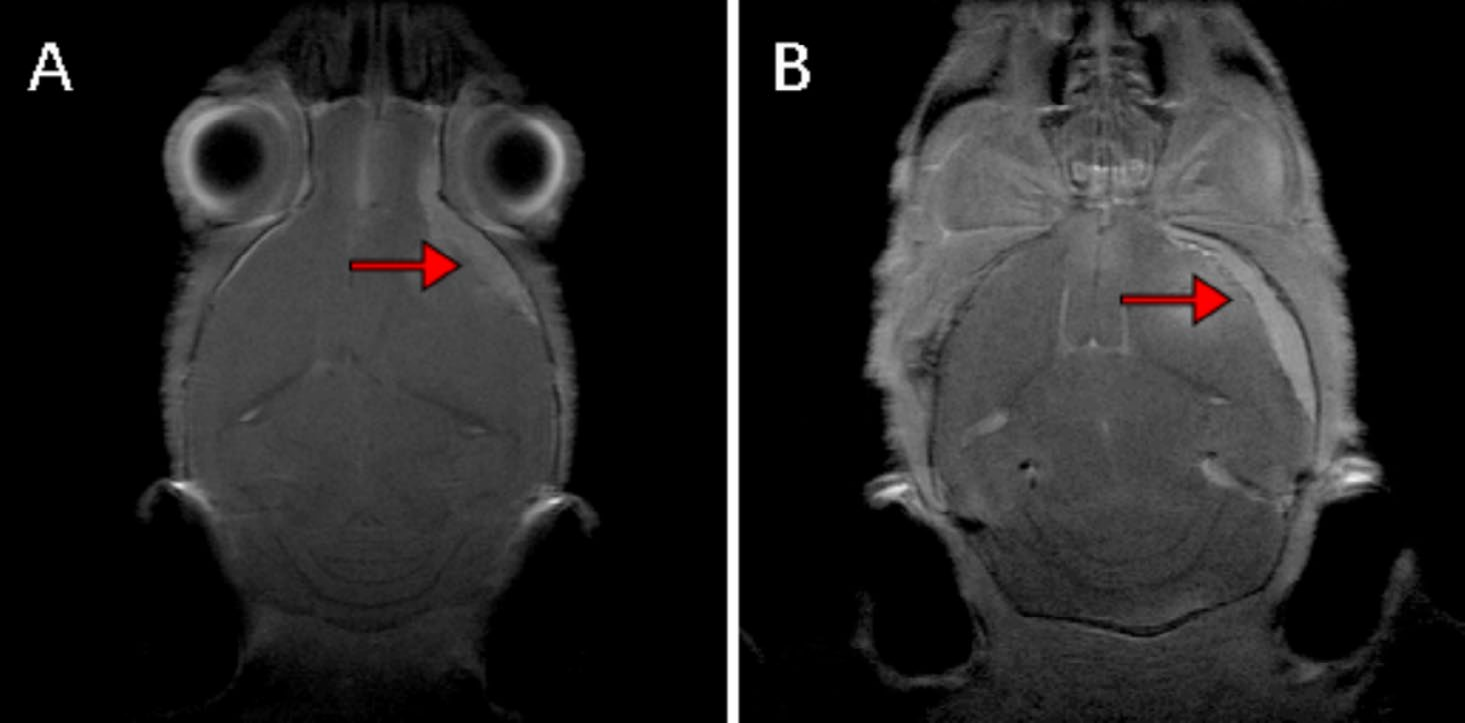
Detection of meningioma formation in mice by brain MRI scan (red marks showed tumor formation located at skull base in mice).

After 10 months' observation, five mice were sent for brain magnetic resonance imaging (MRI) scans, in order to detect intracranial tumor growth. This study was conducted on a Bruker 7.0 T scanner (Bruker BioSpin GmbH). After MRI scanning, tumor formation could be displayed in MRI images (Figure 5).

### 3.5 Histology and Immunohistochemistry

**FIGURE 6:**
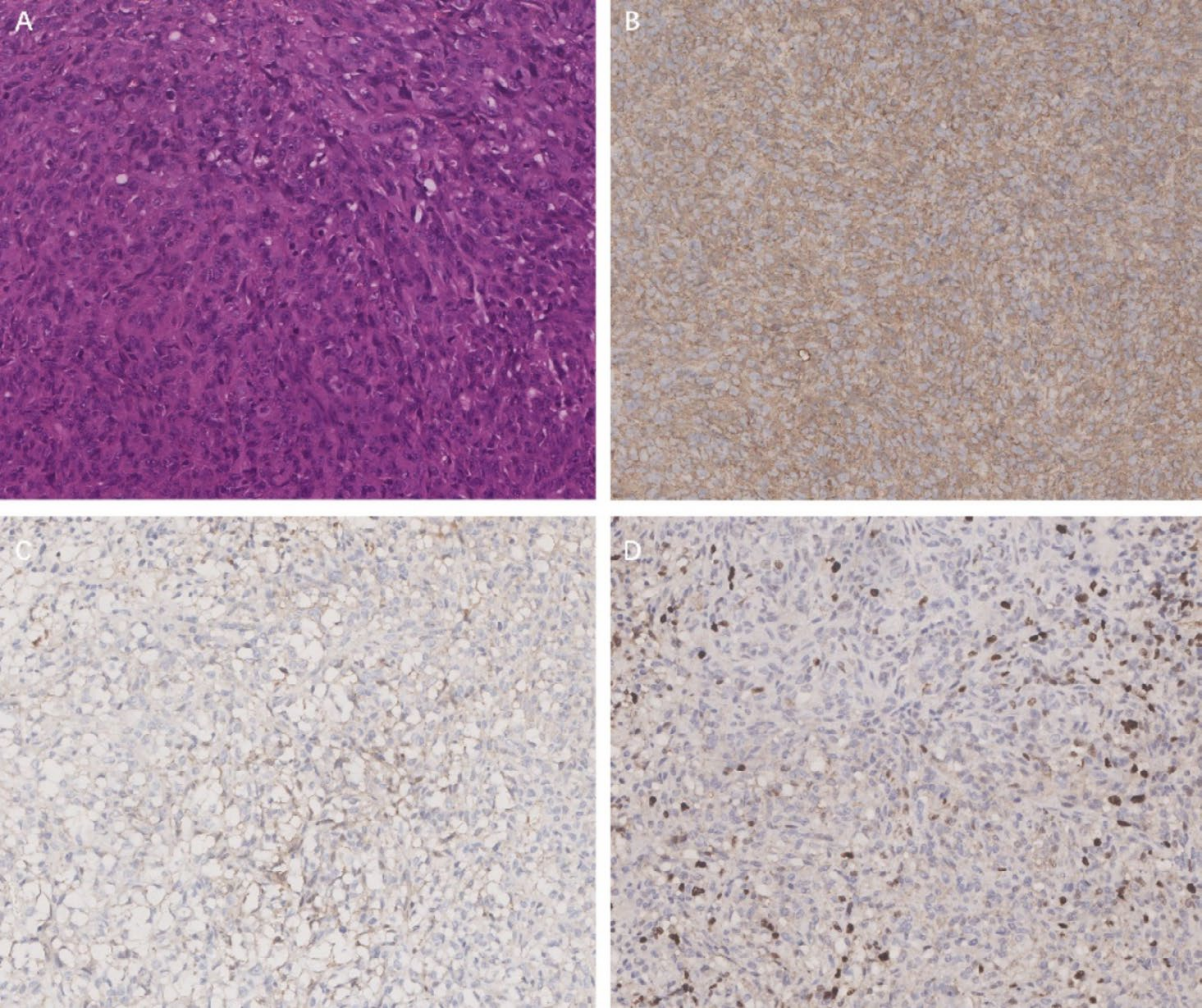
Pathological analysis revealed atypical meningioma features of the tumor sample. (A) H&E staining of the sample, X20, (B) positive for Vimentin, X20, (C) positive for EMA, X20 (D) Ki‐67 index, X20.

Finally, the remaining nine mice tumor specimens were processed for histological examination via standard hematoxylin and eosin (HE) staining and also examined for EMA, vimentin, and Ki‐67 (as detected by MIB‐1 antibody) via immunohistochemical (IHC) staining (Figure 6). Six mice had benign meningioma subtypes similar to human meningioma, one mouse had atypical meningioma, one mouse had malignant meningioma, and one mouse had sarcoma.

## 4 Discussion

Meningiomas, which are thought to arise from the meningeal cells of the brain, are classified into three WHO grades and 15 histological subtypes [11, 12]. Surgery remains the optimal treatment for benign meningiomas but is often insufficient in providing control for grade II or grade III meningiomas, as these tumors display aggressive biological features, including high proliferation activity and infiltrative growth. These cases often require adjuvant radiotherapy or radiosurgery in order to decrease the risk of recurrence [13, 14]. A number of medical treatments have been proposed for these refractory meningiomas and tested for effects, including clinical trials (NCT02523014, NCT03071874, NCT02831257, and EORTC‐1320), but they have shown only limited efficacy.

Genetic alterations of the *Nf2* gene are present in 60% of sporadic meningiomas regardless of grade, suggesting their initiating role in meningeal tumorigenesis; meanwhile, *CDKN2A* (*p16* ^*INK4a*^), p14^ARF^, and *CDKN2B* (*p15* ^*INK4b*^) are thought to be responsible for progression to higher grades of meningioma. Preclinical meningioma animal models have been used in an attempt to mimic the genetic and biological alterations found in human meningioma. This can help to clarify the mechanisms of tumorigenesis (e.g., cell or tissue of origin) [15, 16], progression and initiating events, and to assess the efficacy or toxicity of established or newly developed medical treatments for refractory meningiomas [17].

The current meningioma animal models include xenograft models (orthotopic or heterotopic) with implantation of human cell lines or tumor stem cells [12, 18, 19] or patient‐derived tumors/organoids [20, 21, 22], genetically engineered mouse models (GEMMs), and syngeneic allograft models [23, 24, 25]. With regard to GEMMs, two techniques, including the Cre‐lox system and the RCAS‐TVA system, are commonly used for the establishment of meningioma mouse models. In our study, with some modifications to the Cre‐lox system, we created a new mouse model of skull base meningioma using *Nf2* gene inactivation and the combined loss of *P15* ^*Ink4b*^, *P16* ^*Ink4a*^, and *P19* ^*Arf*^.

Traditionally, the Cre system has been used for genetically editing the target genes [26]. Here we applied the Cas9 tool to improve the efficiency of tumorigenesis, which was activated by Cre, and then the expressed Cas9 could actively edit the target gene mutations necessary for tumorigenesis. In our study, we successfully bred Cas9 transgenic mice as the background host and first performed in vitro experiments. Primary meningeal cells were obtained from these Cas9 transgenic mice. The lentiviral vector pLentiCre/gRNA, which could block the functions of the four genes *Nf2, P15* ^*Ink4b*^, *P16* ^*Ink4a*^, and *P19* ^*Arf*^, was packaged and co‐cultured with these meningeal cells to infect them. The results showed that the virus could infect these meningeal cells and express Cre, which activated Cas9 expression in these meningeal cells (Figure 1). In addition, we discovered that the editing sites set in the meningeal cells of the four target genes were all mutated (Figures 2 and 3). These results suggest that our strategy can indeed achieve mutations of the four genes in meningeal cells derived from Cas9 transgenic mice.

Based on the above in vitro results, we proceeded to the in vivo experiments. Given the high packaging titer and enduring in vivo effect of AAV, we constructed an AAV vector pAAVCre/gRNA, which could import Cre and the above four gRNAs. The prepared pAAVCre/gRNA was injected into the skull base at the subdural position of P2 Cas9 transgenic mice. We observed the expression of GFP at the injection sites (Figure 4), confirming the role of Cas9 in situ, which was activated by Cre. After 10 months of observation, tumor growth was checked by brain MRI scan (Figure 5), and meningioma samples were analyzed in these mice and confirmed via pathological analysis (Figure 6).

In this study, we successfully created a Cas9 mouse model of skull base meningioma initiated with a genetic defect in *Nf2* and the combined loss of *P15* ^*Ink4b*^, *P16* ^*Ink4a*^, and *P19* ^*Arf*^. Animal models based on such a strategy could provide useful tools for investigating the pathogenesis of meningioma, clarifying its mechanisms of progression, and developing chemical interventions, which are currently lacking [27, 28].

However, certain limitations to this study should be acknowledged. First, the financial costs for the acquisition and breeding of these Cas9 mice, and periodic time for tumor growth are burdensome. A relatively long‐term period (10 months in our study) for tumor growth is required, especially for grade I meningioma. Second, this type of mouse meningioma model occasionally results in nonmeningeal tumors, which may arise from the mesoderm tissue and may cause death. Indeed, we detected one sample with sarcoma in our study. Third, due to insufficient resources, we did not divide the *Nf2, P15* ^*Ink4b*^, *P16* ^*Ink4a*^, and *P19* ^*Arf*^ genes into different animal groups. Nonetheless, the combined role of the four genes was associated with tumor growth in Cas9 mice. In the study, we conducted primarily experiments, which for the first time confirmed the successful establishment of a skull base meningioma model in Cas9 mice. However, more elaborate studies are needed that can fully utilize this model in order to clarify the pathogenesis of meningioma and screen for new medical therapies for refractory meningiomas in the future.

In summary, we developed a Cas9 mouse model of skull base meningioma initiated with a genetic defect in *Nf2* and the combined loss of *P15* ^*Ink4b*^, *P16* ^*Ink4a*^, and *P19* ^*Arf*^ in this study that can provide a new tool for investigating the pathogenesis of meningioma and aid in devising chemical treatments for patients with this disease.

## Conflicts of Interest

The authors declare no conflicts of interest.

## Data Availability

The data that support the findings of this study are available from the corresponding author upon reasonable request.
